# The southernmost *Errina antarctica* hydrocoral savannah in Patagonian waters

**DOI:** 10.1038/s41598-024-60207-2

**Published:** 2024-04-26

**Authors:** Ana De la Torriente, Ingrid M. Espinoza-León, Lorena A. Valenzuela-Lobos, Ana Antolinez, Alberto Serrano

**Affiliations:** 1 Instituto Español de Oceanografía, Centro Oceanográfico de Santander (COST-IEO), CSIC, Promontorio San Martín s/n, 39004 Santander, Spain; 2 Fundación Rewilding Chile, Puerto Varas, Chile

**Keywords:** Marine biology, Biodiversity, Conservation biology

## Abstract

Marine animal forest (MAF) are animal-dominated megabenthic communities that support high biodiversity levels and play key roles in ecosystem functioning. However, there is limited data available in Patagonian waters related to the presence of these vulnerable benthic communities. We report a monospecific MAF of *Errina antartica* in Angostura Tomms, which represents the southernmost known living MAF of this species. With coverages reaching up to 28.5% of the substrate from 1.23 m to, at least, 33 m depth is the shallowest stylasterid assemblage described worldwide to date. The size of the colonies ranged from 0.14 to 15.8 cm, with small colonies (< 10 cm) being the most abundant (99%). We hypothesize that this MAF might correspond to a recent colonization of a space, extending its distribution range towards shallower areas or it could be an assemblage formed at the limit of the species’ distribution in which the environmental conditions are not optimal for the major development of the colonies. Additionally, results showed that habitats structured by three-dimensional sessile invertebrate such as *E. antarctica* showed higher values of species richness and alpha diversity than non-biogenic habitats. Analyses were based on 297 photos taken at 22 different sites in the western Strait of Magellan, along vertical transects from 5 to 25 m depth. Our study highlights the importance of the benthic communities existing in Patagonian waters, evidencing the need to act actively to ensure their maintenance.

## Introduction

Marine animal forest (MAFs) are recognized as complex animal-dominated megabenthic communities that support high species richness and biodiversity levels^1–3^. The high densities that erect habitat-forming species (HFS) can reach in these submerged forests generate a high structural complexity that shapes the biotope available to many other species and provides microniches used by a diverse associated benthic fauna^2,4,5^. It is clear that these habitats play a key role in ecosystem functioning such as maintenance of wildlife, enhancement of a suitable environment for spawning, breeding and feeding areas for fish and invertebrate species, availability of refuge for endangered species and favoring benthopelagic coupling^4,6^. Additionally, they provide essential ecosystem services worldwide, such as potential carbon sinks, sustainability of fisheries, ecotourism and recreational activities for humans^4^. However, many aspects related to their distribution, population dynamics, trophic ecology, and functionality, remain poorly known.

MAFs can be found in all oceans, from shallow to deep waters, and can be dominated by a great diversity of sessile and suspension feeders such as corals, sponges, ascidians, brachiopods, polychaetes and mollusks^2,7–9^. In Chile, various MAFs have been described, both dominated by multiple species such as mussel banks (*Mytilus chilensis* and *Aulacomya atra*) or cold-water stony coral reefs (*Desmophyllum dianthus*, *Caryophyllia huinayensis* and *Tethocyathus endesa*) and by a single species such as polychaete fields (*Chaetopterus variopedatus*), gorgonian forest (*Thouarella* sp.) or ascidians fields (*Sycozoa sigillinoides*)^2^. However, among these forest formers, the capacity of hydroids as HFS has been underestimated worldwide, and, therefore, its importance in enhancing local biodiversity underappreciated^10^. Nevertheless, hydrozoans are capable of modifying environmental conditions by affecting water movement, providing space for settlement and enhancing habitat complexity^10–13^, especially those perennial hydrozoans that give rise to year-round animal forests. Stylasteridae, as hydroids that are always present in their erect form with a calcareous skeleton typically branched and arborescent, facilitate the formation of three-dimensional environments^4,14^. Several species belonging to this family of hydroids have been reported appearing in high abundances in different parts of the world. For example, the extensive settlements of *Errina aspera* at 80–130 m depth in the Messina Strait^11,15^, several populations of *Errina novaezelandiae* in New Zealand’s fjords^16^, a dense aggregation of *Stylaster flabelliformis* in Reunion Island (Sainte-Rose) at 75–100 m depth^17^ and the shallow-water aggregation of *Stylaster californicus*^18^.

In continental Chile, of the 314 described species of this calcified stylasterids^19^, only two species, *Allopora profunda* and *Errina antarctica*, have been recorded, both in Patagonian waters^20,21^; however, only forests of *Errina antarctica* have been documented^2,21^. The distribution of this species in that region includes depths from 10 to 120 m, from northern (south of Chiloe) to southern Patagonia (Strait of Magellan). Despite being a common component of benthic communities in Patagonian channels^2^, its occurrence in high densities forming a MAF has been confirmed only in the Copihue Channel-Madre de Dios Archipelago, in Central Patagonian Zone^21^, on hard substratum below 10 m depth. This structural keystone species is distributed, in addition to Chilean Patagonian waters, in the Falkland Islands, and the Scotia Arc (Burdwood Bank)^20^.

This slow-growing, sessile and limited dispersal potential hydroid with a calcified skeleton easily damaged is particularly vulnerable to disturbance^16^. The presence of reef-like formation of this species has been linked to pristine or scarcely disturbed habitats^10,21^, so information related to its distribution and population dynamics is highly relevant for designing marine management and conservation programs. As a sentinel habitat, their physical reduction or loss reflects the occurrence of unfavorable environmental conditions caused by both natural changes and/or anthropogenic threats. This is the specific case of the *Errina antarctica* forest found in 2006 in the Copihue Channel, which was subsequently found wiped out in 2013 for unknown reasons and of which only hydrocoral rubble remains accumulated on the bottom^22^.

The removal of MAF causes the homogenization of the seafloor habitats^23^ and ultimately alter the ecosystem functioning of benthic habitats^24^. By contrast, biogenic habitats, characterized by higher diversities and functional redundancies, are more buffered environments, less susceptible to changes and species loss^25,26^ and more able to maintain ecosystem functioning^3,27,28^, highlighting the need for their effective protection.

Chilean Patagonian is characterized by highly complex geomorphology and hydrographic conditions with strong gradients that create microenvironments, sustaining a diversity of unique and vulnerable benthic communities^2,29,30^. Located on the southeastern border of the Pacific Ocean, this region has an extensive coastline of ca. 84,000 km, and it is composed of many islands, fjords, and channels^31^. Scientific exploration of this region has been limited due to its remote location and extreme environmental conditions most of the year, characterized by low temperatures, high wind speeds, strong storms and high rainfall (around 3000–4000 mm per year on the coast and more than 7 000 mm per year in certain inland areas)^32,33^. Consequently, benthic communities are largely unknown and likely contain numerous undiscovered species and unknown animal forest locations. This lack of knowledge of the marine ecosystem hampers our ability to design effective and suitable long-term sustainable management programs and meet international targets.

Among these objectives, it is proposed to conserve at least 30% of the seas and oceans by 2030 (CBD/COP15/2022). While conservation efforts in Chile represent important protection advances that have materialized in the designation of 42% of Chile’s exclusive economic zone (EEZ) as marine protected areas, less than 1% of the channels, fjords, and the continental shelf and slope in Patagonia are included in these areas, illustrating a lack of representation of this marine ecosystem in Chilean conservation policies. This region, however, is threatened by pollution, eutrophication, harvesting, particle sedimentation, salmon and bivalve aquaculture, introduction of invasive species, ocean acidification or global warming^34^. This evidences the urgent need for action to meet a compromise between conservation of biological communities and sustainable maintenance of the economy of southern local populations.

This study reports on the discovery of a new *Errina antarctica* forest and improves the knowledge about the distribution of these MAFs in southern Chile. Using a photographic methodology taken by scientific scuba diving, its role in the benthic community is studied through the analysis of its distribution, morphometric parameters of the colonies as well as the diversity of associated invertebrate fauna. This information is crucial to improve the baseline knowledge of the Patagonian marine ecosystem for their effective protection and sustainable use development.

## Methods

### Study area

The Strait of Magellan is the most important channel in southern Patagonia that connects the Pacific and Atlantic oceans and separates the southern part of South America from Tierra del Fuego and the Fueguian archipelago. It is about 550 km long and is characterized by the presence of several micro-basins along its length^35^. The area is influenced by saline water masses from the Pacific, Atlantic and Southern oceans that mix with freshwater from rainfall, river inputs and snowmelt, generating an estuarine system^31^.

**Figure 1:**
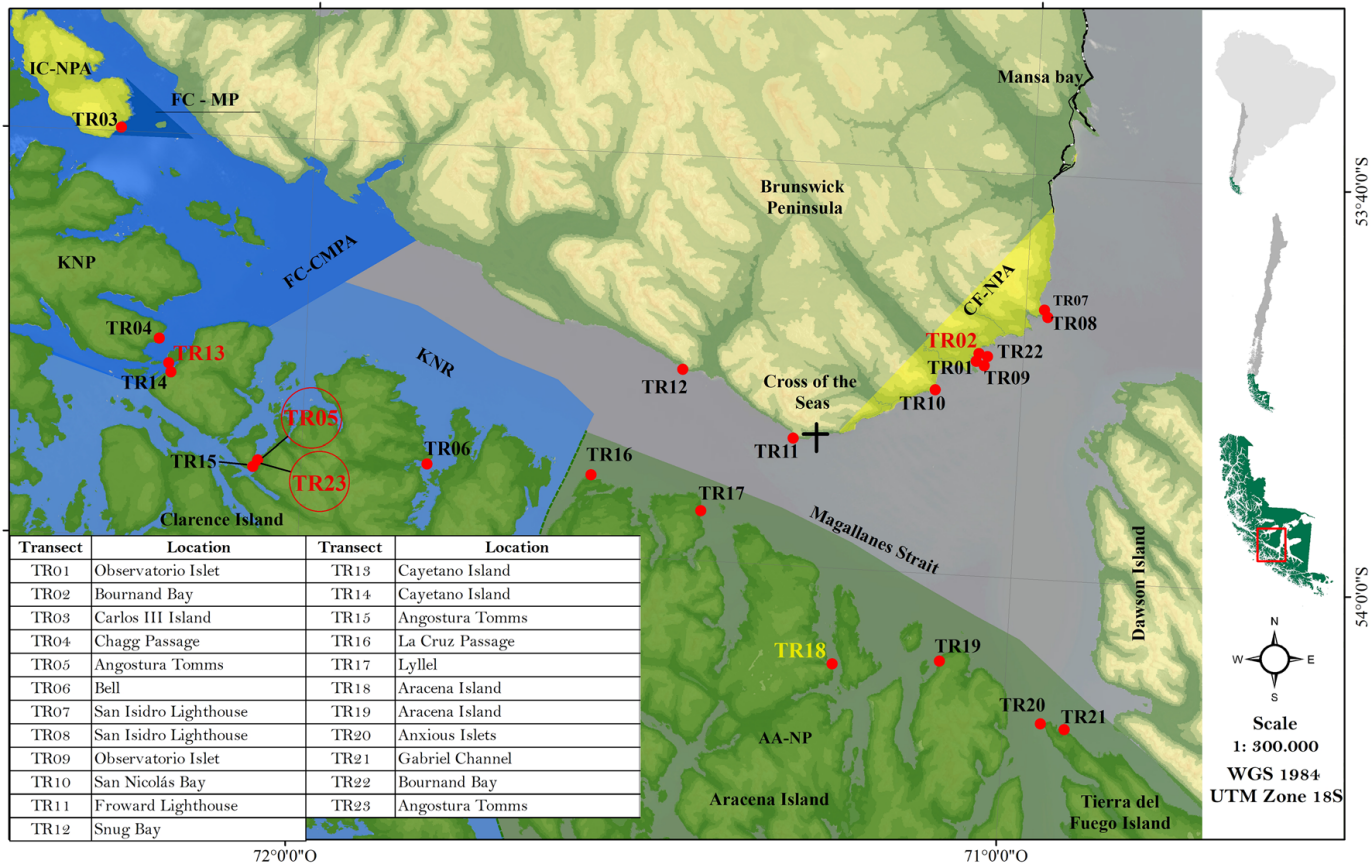
Map of the study area in the central micro-basin of the Strait of Magellan showing the scuba diving sampling stations (red dots). Nearby protected areas are displayed in different colors. Inset shows the location in the southern South America. Sampling sites with *E. antarctica* are shown in burgundy, while sites where *C. variopedatus* was recorded, in mustard. The circle marks the sites where a high abundance of *E.antarctica* colonies forming a MAF was found. Data layers: Ministry of the Environment, Government of Chile: https://simbio.mma.gob.cl/AreaProtegida/IndexDesignaciones/1161, and National System of Protected Wild Areas of the State, Ministry of Agriculture, Government of Chile: https://idembn.bienes.cl/catastro/map/19?zoom=8.1918669962107&center=-7946632.234924241%2C-7136628.728579631. Using: ArcGIS for desktop advanced [GIS]. Version 10.8.2, Esri.

Our study focused on sampling an area located in the central micro-basin, specifically between Cape Froward and Carlos III Island (Fig. 1). While no management measures have been implemented in part of the study area, some sectors are included in one of the four MPAs that border the study area: (i) the Francisco Coloane Coastal and Marine Protected Area of Multiple Uses (FC-CMPA), (ii) the Kawésqar National Reserve (KNR), (iii) the Francisco Coloane Marine Park (FC-MP), and (iv) the Alberto de Agostini National Park (AA-NP). The first two allow multiple uses while FC-MP and AA-NP are no-take zones. In addition, the study area is adjacent to three terrestrial protected areas: the Cabo Froward National Protected Asset (CF-NPA), the Carlos III Island National Protected Asset (IC-NPA) and the Kawésqar National Park (KNP).

### Sampling and data analysis

**Figure 2:**
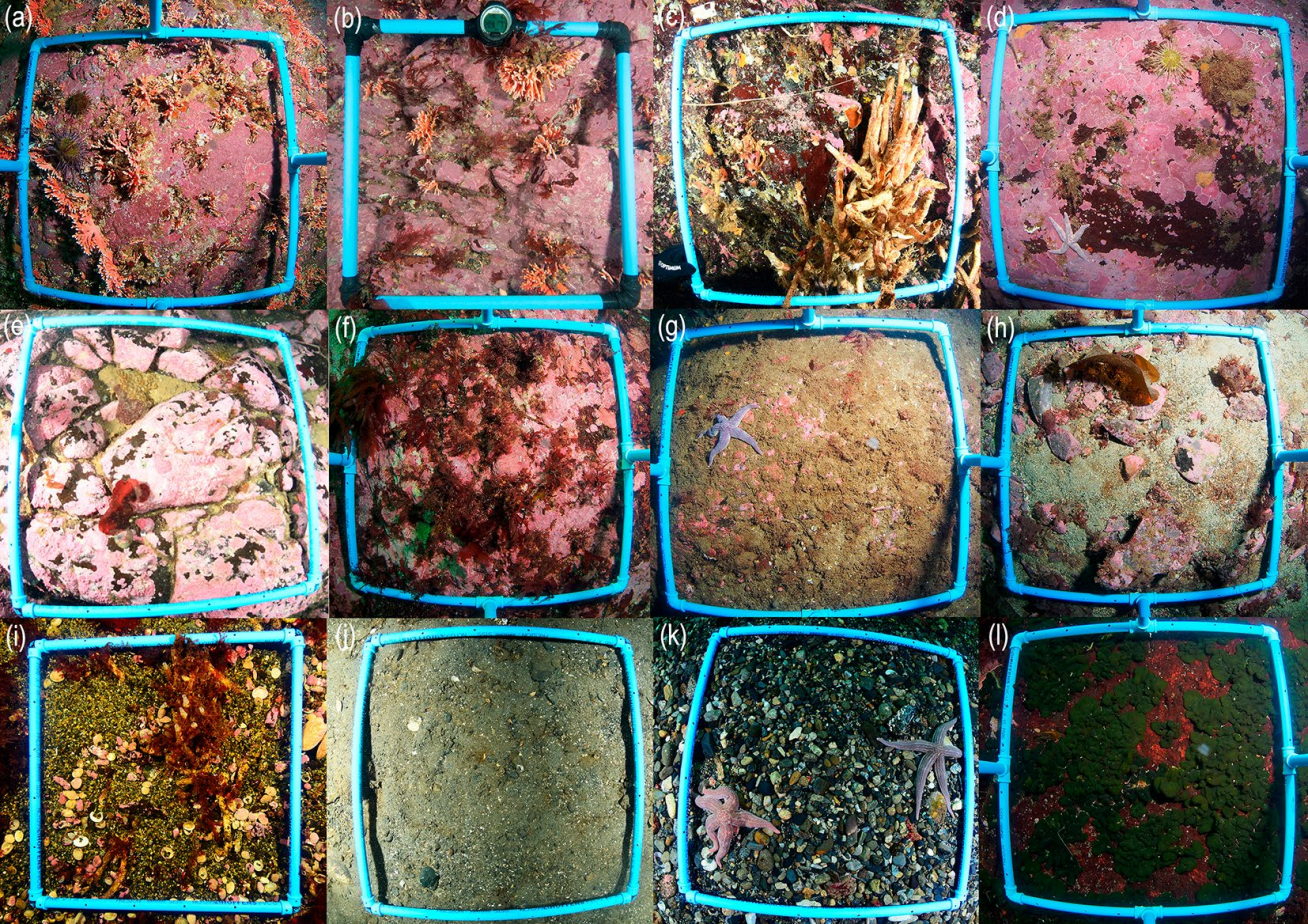
Classification of benthic habitats according to the type of substrate, the presence of HFS and the presence of sedimentary or biological coverage: (**a**,**b**) Hab1-Rocky bottom with *Errina antarctica*, (**c**) Hab2-Rocky bottom with *Chaetopterus variopedatus*, (**d**–**f**) Hab3-Rocky bottom with crustose coralline algae coverage, (**g**) Hab4-Rocky bottom with sedimentary coverage, (**h**) Hab5-Mixed sandy-rocky bottom, (**i**,**j**) Hab6-Gravel and/or sandy bottom with shell material, (**k**) Hab7-Pebbles bottom, and (**l**) Hab8-Rocky bottom with algae *Codium dimorphum* coverage.

Two scuba-diving expeditions were carried out in the western area of the Strait of Magellan in April and July 2021, between Carlos III Island and Cape Froward. Photographs were taken along vertical transects from 5 to 15 m depth at 22 sites. At some sites, when conditions permitted, transects were extended up to 25 m depth (Supplementary Table St1). The locations of the transects were randomly chosen along both coastlines and channels on the western side of the Strait of Magellan (Fig. 1). Each transect was sampled every 5 m depth and consisted of photographing a 50 × 50 cm grid. Between four and five replicates were taken per depth, except for transect TR01 in which only three replicates were taken at 5 m depth. An additional scuba-dive was conducted in August 2022 at the *Errina antarctica* assemblage occurrence site from surface to 20 m to complement the MAF-specific information, adding seven more grid-photographs to the analysis. The depth of all photographic replicates was checked with respect to the lower mean low tide (LMT), considering the tidal coefficient of each date and time; as only in one station the difference was 2 m and in 78% of the stations the tidal difference was less than 1.6 m, no depth corrections were made. A total of 297 photos were obtained for subsequent analysis. To analyze the distribution of *Errina antarctica* at different depths, its coverage (as percentage of the frame’s area) and the abundance of colonies were calculated from the 18 photos where the presence of this species was recorded. Because the number of samples in each group was not high, we cannot ensure that the data met parametric data requirements, therefore Kruskal–Wallis test^36^ was applied on both metrics to verify the significant difference between depths. Image analysis also allowed their size-structure to be analyzed, based on their maximum length. Colonies were classified into four categories (0 < 2 cm; 2 < 5 cm; 5 < 8 cm; and ≥ 8 cm) and their relative frequency assessed for each depth range. Additionally, at the *Errina antarctica* assemblage site, random photographs and a continuous video along a vertical transect from 33 to 17 m were taken to complementarily characterize the species associated to this MAF based on the organisms sharing the habitat with the stylasterid. Of this added information, only close up pictures and frames where at least one colony of the species was present were selected. Furthermore, a remotely operated vehicle (ROV) dive was conducted in August 2023 to obtain more data on the occurrence of this MAF in deeper areas, reaching down to 47 m depth. To study the faunal composition associated with the assemblage structured by *E. antarctica* and compare it with the rest of the communities found in the study area, each photo was characterized by the type of substrate (rocky, pebble, gravel, shell material, sand), by the occurrence of HFS (only *Errina antarctica* and *Chaetopterus variopedatus* were found structuring the benthic assemblages in some of the sampling sites) and by the presence of some sedimentary, crustose coralline algae or green algae *Codium dimorphum* coverage. Based on the different combinations of these data existing in the study area, each photo was classified into the following habitat types, both biogenic and non-biogenic (Fig. 2): Hab1-Rocky bottom with *Errina antarctica*, Hab2-Rocky bottom with *Chaetopterus variopedatus*, Hab3-Rocky bottom with crustose coralline algae coverage, Hab4-Rocky bottom with sedimentary coverage, Hab5-Mixed sandy-rocky bottom, Hab6-Gravel and/or sandy bottom with shell material, Hab7-Pebbles bottom, and Hab8-Rocky bottom with algae *Codium dimorphum* coverage. The latter was eliminated from the subsequent analysis because the algae did not allow the organisms to be seen, covering practically the entire photo.

All megabenthic (> 2 cm) invertebrate organisms contained in the grid were identified to the lowest possible taxonomic level and quantified in abundance terms (number of individuals or colonies). When there was no confidence regarding the species identity of individuals, specimens were identified to genus, family or morphotypes (operational taxonomic units or OTU) to overcome taxonomic inconsistencies. Nomenclature was standardized based on the World Register of Marine Species database (WoRMS 2023^37^).

A quantitative analysis of the species composition of the benthic communities was performed. Taxonomic species richness and alpha diversity of the MAF of *E. antarctica* was compared with those found in the rest of the habitats identified in the study area. For this purpose, we used only photos taken at 15, 20 and 25 m at all sampling sites, coinciding with the maximum density of *E. antarctica*. Photos of transects TR04 and TR20 were not selected since it was only possible to take photos at 5 and 10 m depth. Preliminary analyses showed that the effect of year was statistically non-significant and therefore, samples from both years were used. Once the samples without any species and the only sample of habitat eight in the depth range of this analysis were removed, the data set for this analysis was composed by 112 photo-samples. Both, species richness (S, as average OTU per sample) and Shannon–Wiener index (H’, using log2^38^) were calculated using the “vegan” package^39^. The two habitat-forming species of the two existing biogenic habitats in the study area, *C. variopedatus* and *E. antarctica*, were eliminated from this last specific analysis to prevent their high abundances from masking their effect on diversity of benthic species associated with each habitat.

One-way analysis of variance (ANOVA) was used to estimate significant differences in species richness and Shannon–Wiener index among habitats. The ANOVA assumptions were tested and corroborated graphically plotting residuals vs. fitted values and normality of residuals^40^. When ANOVA showed significant differences, “post-hoc” Tukey’s honestly significant difference (HSD) tests were performed to determine which habitats were significantly different from the others^41^. In addition, species accumulation and k-dominance curves for each habitat were plotted to express how evenly individuals are distributed among the different species within the habitats. To test for differences in species composition among habitats, PERMutational ANalysis Of VAriance (PERMANOVA) using Bray Custis distance was performed. This analysis was applied using “vegan” package^39^, with p-values for the pseudo-F test statistic based on 999 permutations. The taxonomic composition of all habitats was examined at phylum level based on their relative species richness and their abundance of individuals or colonies. Previously, the abundance of *E. antarctica* and *C. variopedatus* were eliminated from habitats one and two respectively. Additionally, a similarity percentage analysis (SIMPER^42^, cut-off, 95%) on the abundance data matrix was applied to examine the dissimilarity between the habitats concerning taxonomic composition, and additionally, to identify the main taxa typifying each habitat and therefore, contributing most to the similarity within each habitat.

## Results

**Figure 3:**
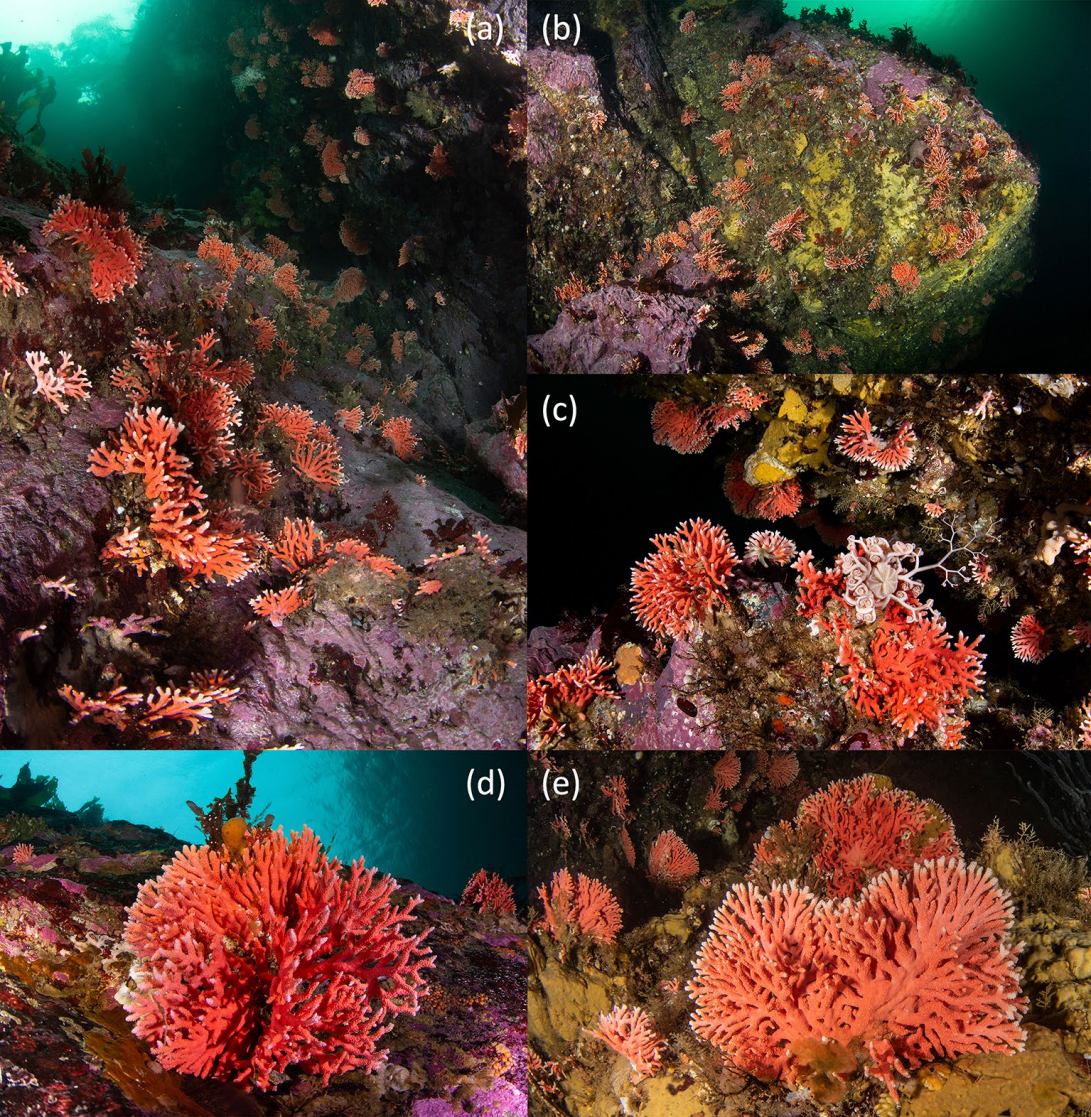
Representative photographs of the *Errina antarctica* MAF and colonies found in Angostura Tomms, very close to the Strait of Magellan. The colonies grow both on vertical rocky walls (**a**-**c**) and on horizontal hard bottoms (**d**,**e**), forming a reef-like formation. Two extreme growth forms of *E. antarctica* were recorded: (**d**) densely branched multiplanar colony and (**e**) two-dimensional fan-shaped.

Colonies of *Errina antarctica* were found at four of the 23 transects or sampling sites, located in Bahía Bournand (Cabo Froward; TR02), Punta León (Barbara Channel; TR13) and Angostura Tomms (NW Clarence Island, TR05 and TR23) (Fig. 1). In two of the sites (Bahía Bournand and Punta Leon) isolated colonies were documented and only in transects carried out in Angostura Tomms, a MAF of *Errina antarctica* was found (Fig. 3).

The upper limit recorded of its bathymetric distribution was 3.1 m depth, which corrected with the tidal coefficient, places the forest at 1.23 m with respect to the lower mean low tide (LMT). The lower limit was not reached due to being below diving depths, however, although the MAF of *E. antarctica* was quantitatively studied only down to 25 m, the scuba-diving video footage showed its existence at least down to 33 m depth (Supplementary Video Sv1) and the ROV video down to 46.5 m depth (Supplementary Video Sv2).

At this site, the coverage of this stylasterid ranged from 7.34 to 28.54% of the substrate (mean = 14.24 ± 5.4%) and the density of the species ranged from 36 colonies/m^2^ to 436 colonies/m^2^, reaching that maximum value at 20 m depth. The mean density of the assemblage was 205 ± 126 colonies/m^2^.

**Figure 4:**
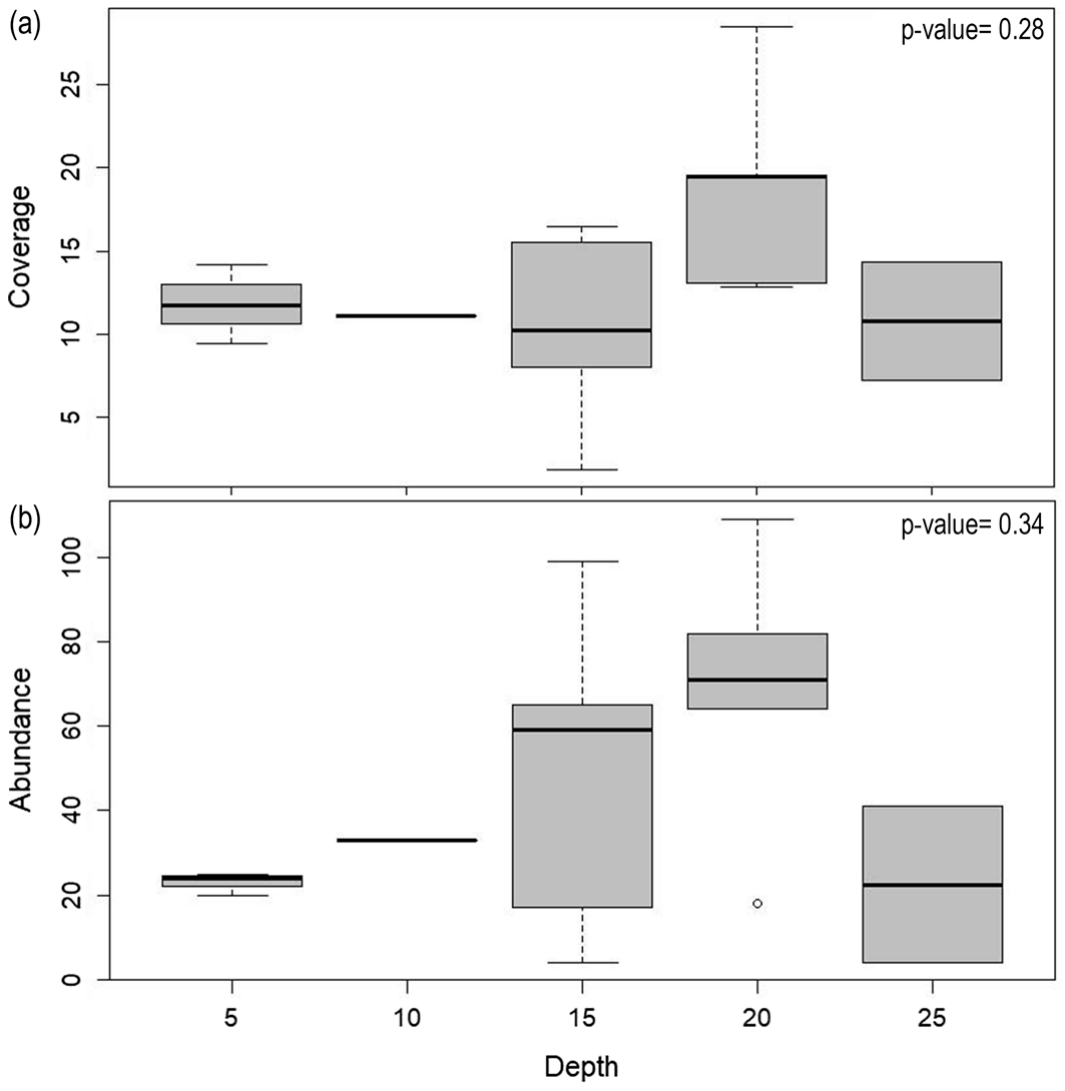
Depth distribution of *Errina antarctica* using (**a**) coverage (%) and (**b**) abundance of colonies. Plots indicate median and standards errors while boxes indicate the 25th and 75th quartiles. Statistically significant differences (p < 0.05) based on Kruskal–Wallis test were not found.

No statistically significant differences were found in the distribution of coverage (%) and abundance of colonies of *Errina antarctica* with depth (Fig. 4). However, a trend towards an increase in the density of colonies was observed at depths of 15 and 20 m, using both the percentage of surface covered and the abundance of colonies, the trend being more pronounced when using the latter parameter.

**Figure 5:**
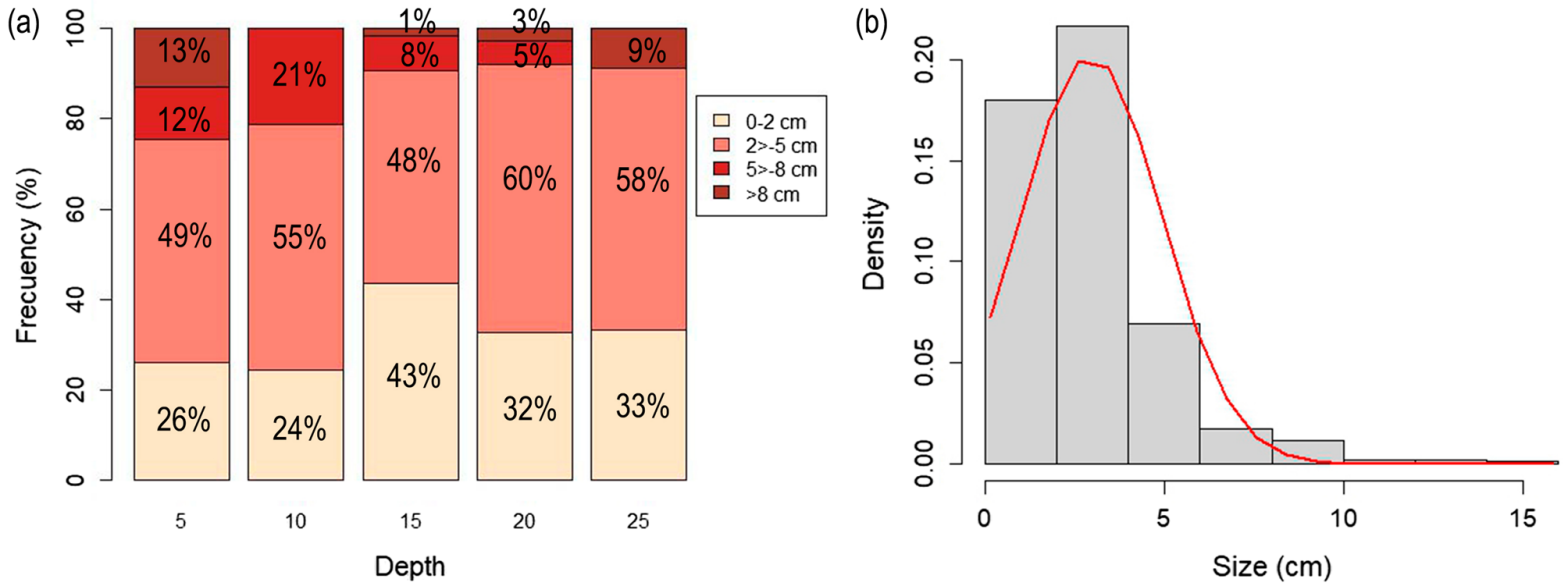
Size structure of the marine animal forest (MAF) of *Errina antarctica* in the Angostura Tomms population: (**a**) relative frequency of each size category at different depths; and (**b**) histogram showing the size-distribution of the colonies and the normal curve (red line).

Different growth forms of *E. antarctica* were recorded: two-dimensional fan shaped, three-dimensional bushy form with few branches and large densely branched multi-planar colonies (Fig. 3). The minimum and maximum sizes recorded were 0.14 and 15,84 cm (mean = 2.96 ± 1.97), and size-structure analyses showed a pattern in which no significant differences were found along depth, although the relative proportion of larger colonies was found at 5 and 10 m depths (Fig. 5a). Size-distribution was unimodal and positively skewed, indicating a MAF dominated by small to medium size classes (Fig. 5b).

**Figure 6:**
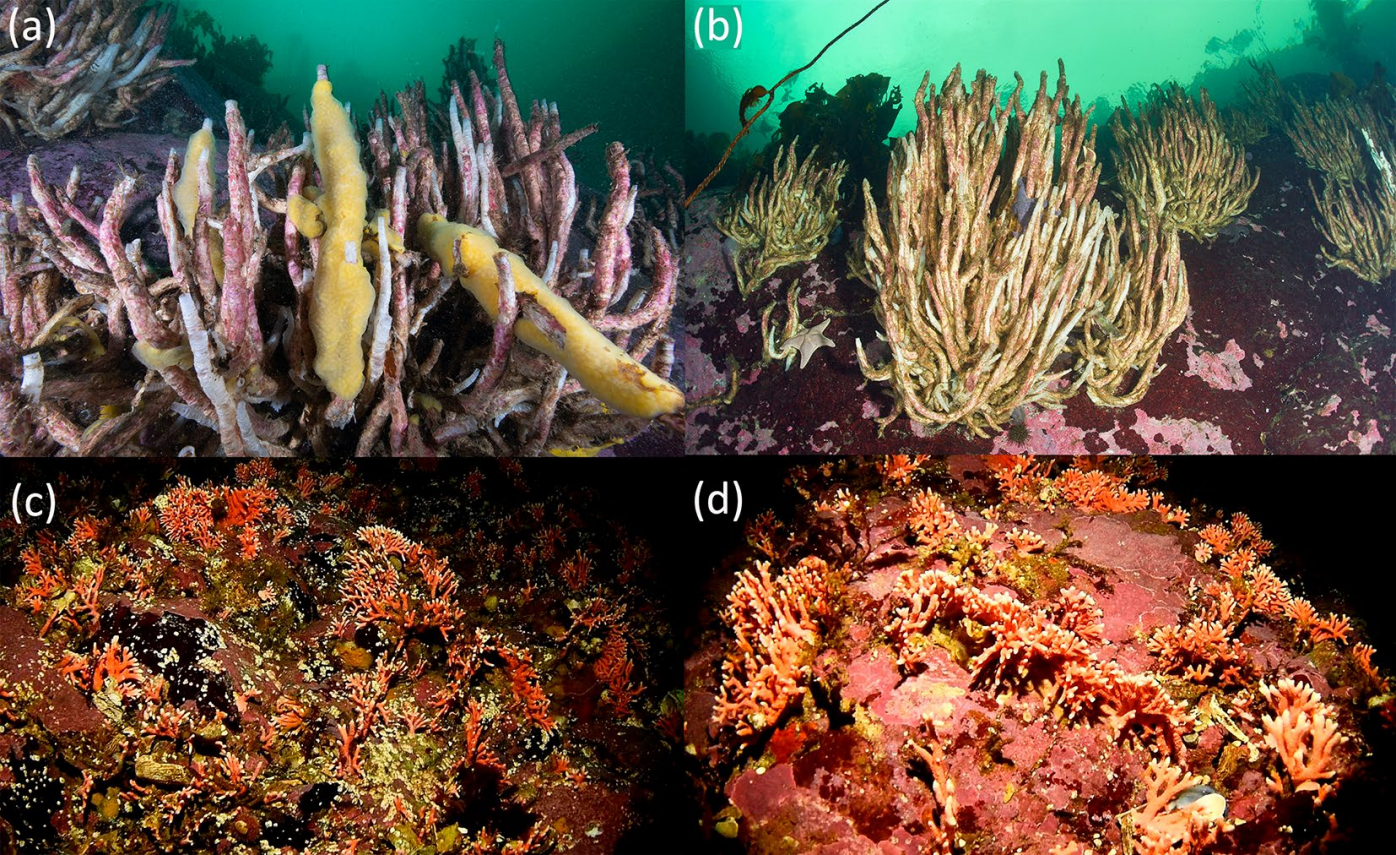
Biogenic communities found in the study area, structured by: (**a**,**b**) *C. variopedatus*, and (**c**,**d**) *E. antarctica.* Photographs (**a**,**b**) were obtained directly by divers while photographs (**c**,**d**) are frames obtained from video footage.

**Figure 7:**
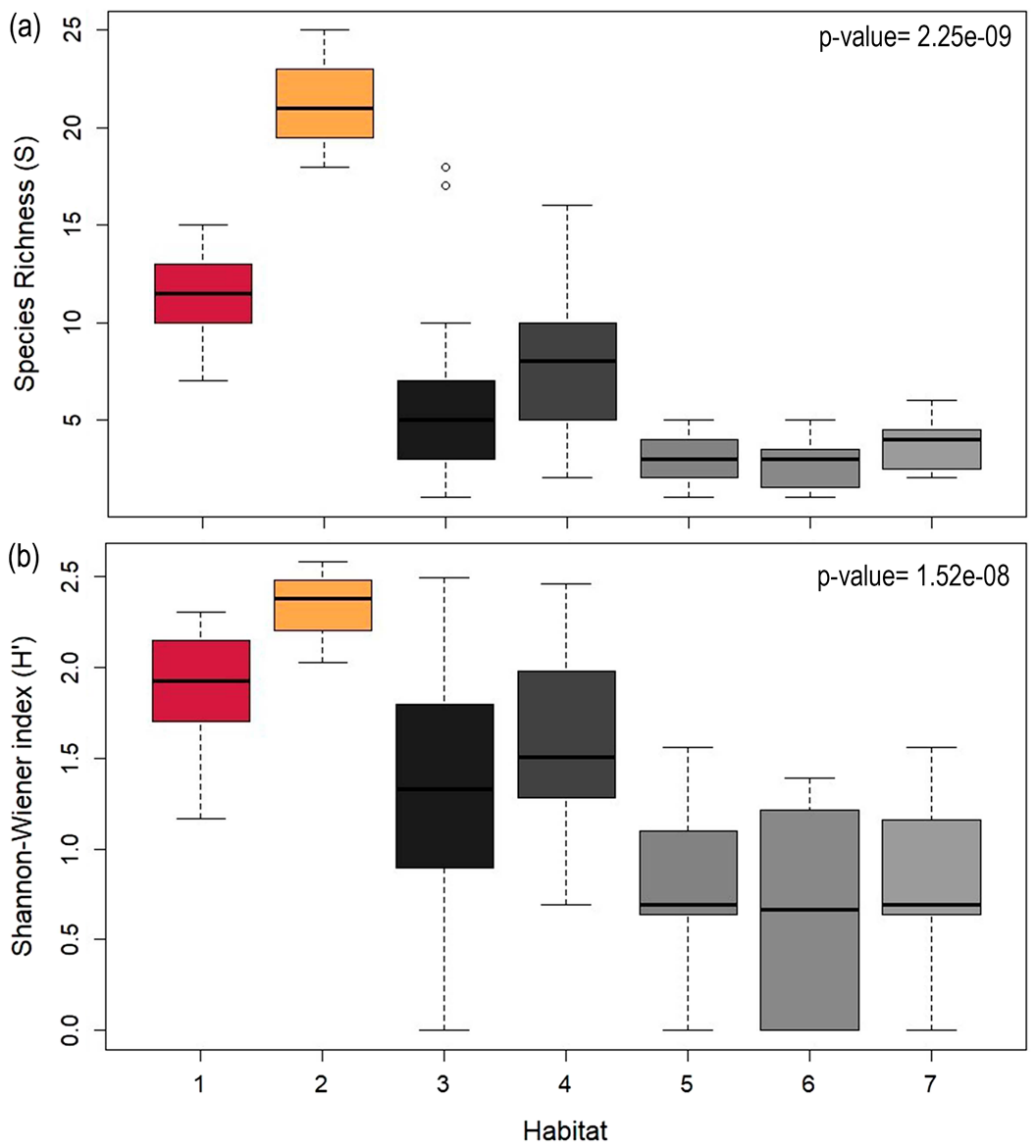
Composition and diversity of invertebrates associated to the different benthic habitats, expressed by different indices as average per sample: (**a**) Taxonomic species richness (S) and (**b**) Shannon–Weaner diversity index (H’). Biogenic habitats are shown in salmon (structured by *Errina antarctica*) and mustard (structured by *Chaetopterus variopedatus*), while non-biogenic habitats are indicated in gray (dark gray for non-biogenic habitats on hard bottoms and light gray for non-biogenic habitats on soft substrates).

**Figure 8:**
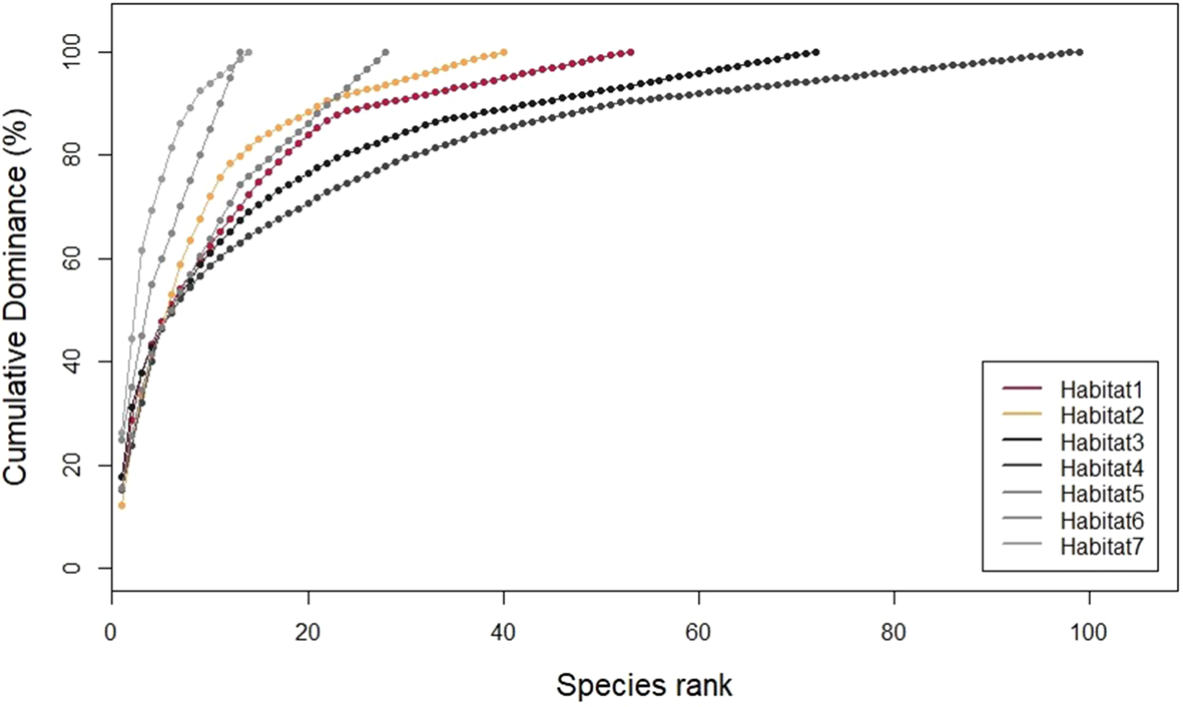
K-dominance curves of cumulative dominance (%) against rank in decreasing order of their abundance for the seven habitats studied. Sample sizes were different for each habitat (N_Hab1_ = 12, N_Hab2_ = 3, N_Hab3_ = 29, N_Hab4_ = 25, N_Hab5_ = 18, N_Hab6_ = 8, N_Hab7_ = 11).

Species richness and alpha diversity analyses based on observed OTUs showed significant differences among habitats (F-value = 42.89; p-value < 0.05 and F-value = 37.7; p-value < 0.05, respectively). The two biogenic habitats structured by HFS, both *E. antarctica* and *C. variopedatus* (Fig. 6), showed significant higher species richness and diversity than those without a structured biogenic component (non-biogenic habitats) (Figs. 7 and 8) as indicated by the post hoc Tukey’s multiple comparison test. Although the values were higher, it is likely that the number of species and alpha diversity values found were underestimated in biogenic habitats. Because some small macrofaunal and meiofaunal species are hidden among the structuring species, they were not exposed for detection through photographs. Similarly, the likely occurrences of some short-lived, fast-growing species or with specific settlement patterns that change from month to month, were neither visible nor counted. Tukey’s multiple comparison test also indicated significant differences between habitats on soft bottoms (habitats five, six and seven) and all the other habitats on rocky bottoms, suggesting that the hard substrate type also determine the existence of greater alpha diversity.

The species accumulation curves (SAC) showed the same trend when species richness among habitats was compared (Supplementary Figure Sf1): at the same sample size, curves showed highest values of species richness in biogenic habitats on rocky bottoms, intermediate values in non-biogenic habitats on hard bottoms and the lowest values in non-biogenic habitats on soft bottoms.

In absolute terms, the habitats that showed the highest species richness values were the non-biogenic habitats on hard bottoms (habitats four and five), as shown by the length of the curves, with the longest ones being those that showed the highest number of species (Fig. 8). The different samples sizes for each habitat explained the different total species richness values shown by each habitat and, therefore, these values should be taken with caution. The plot also indicated that the species composition in all the habitats is characterized by high dominance of a small number of species and low evenness, as reflected by the steep slope of all the different curves. The fact that high-ranking species have much higher abundances than low-ranking species could explain why the Shannon–Wiener Index values were not higher.

**Figure 9:**
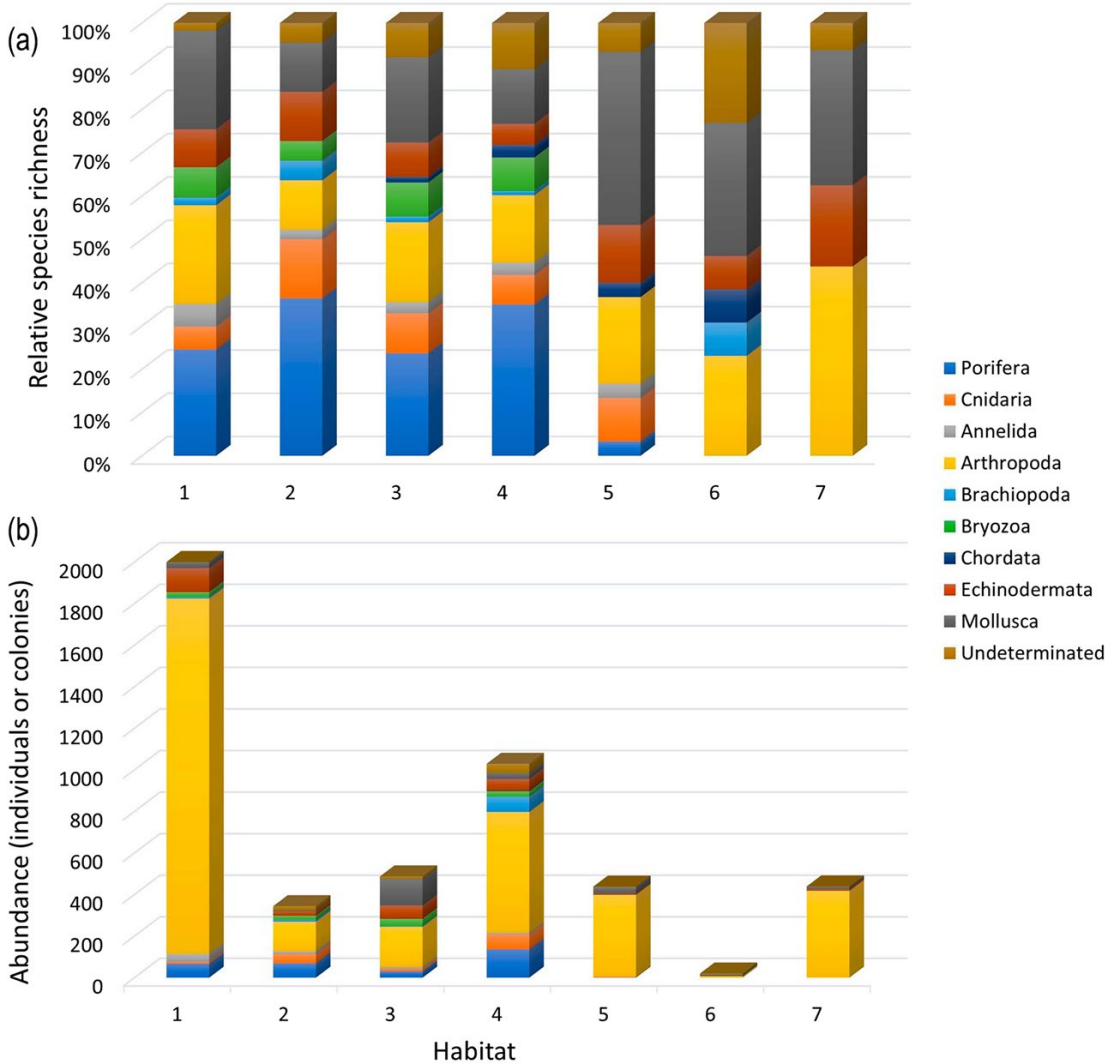
Taxonomic composition of benthic habitats at the phylum level in shown: (**a**) the relative contribution of species richness and (**b**) the total abundance of different groups. MAF of *E. antarctica* on rocky bottoms corresponds to habitat one and that rocky habitat structured by *C. variopedatus* to habitat two; rocky non-biogenic habitats correspond to habitats three and four, mixed sandy-rocky bottoms to habitats five and soft bottoms with gravel and pebbles to habitats six and seven. Sample sizes were different for each habitat (N_Hab1_ = 12, N_Hab2_ = 3, N_Hab3_ = 29, N_Hab4_ = 25, N_Hab5_ = 18, N_Hab6_ = 8, N_Hab7_ = 11).

The PERMANOVA showed differences in the taxonomic composition among the benthic habitats identified in the study area (df = 1, F = 4.4932, p = 0.001). Average between-group dissimilarities were higher than 84.06% in all cases (Supplementary Table St2). Clear differences in the number of species of different taxonomic groups between habitats on soft and hard bottom were found (Fig. 9), while the habitat on mixed sandy-rocky bottoms showed an intermediate composition (Fig. 9a). On rocky habitats, phylum Porifera accounted for between 23.68% and 36.36% of the species richness, while species of this phylum were not present on habitats on soft bottoms. Two other groups that were present in hard habitats but not present in soft habitats were cnidarians and bryozoans, although they contributed with lower relative percentages of species. Mollusk species, however, were found in all habitats, although the relative species richness was higher in soft and mixed bottom habitats, where this group reached the highest relative percentage (31.25% and 40.0% respectively). The same occurred with the species of the phylum Arthropoda, which reached their highest relative contribution (43.75%) on one of the habitats on soft substrate (habitat seven).

In total, we identified 251 unique invertebrate taxa or OTUs from all photographic and video material as organisms being part of benthic communities of the study area. Differences in the number of individuals or colonies among habitats are shown in Fig. 9b, although the total numbers are not comparative since sample sizes were different for each habitat. Arthropoda was the most abundant group, with numbers between 142 (habitat two) and 1719 (habitat one), except in habitat six where the number of individuals of all groups was lower (total abundance = 20 individuals).

Specifically, in the MAF of *Errina antarctica* (habitat one), species belonging to nine different phyla were identified (Table 1). The groups with the highest relative number of species were sponges, cnidarians, arthropods, and mollusks: phylum Porifera accounted for 24.14%, Cnidaria for 22.41% and Mollusca for 20.69% of the total species richness. It is worth noting that the total abundance of this habitat (2009 individuals or colonies—670 ind. or col./m^2^—) is much higher than that of non-biogenic habitats on hard substrate (487 individual or colonies—67 ind. or col./m^2^—and 1029 individuals or colonies—164 ind. or col./m^2^—in habitats three and four respectively), despite having a smaller samples size. The most abundant species identified associated to this assemblage were balanimorphs (Arthropoda) such as *Balanus laevis* and *Elminius kingii*, followed by the sea cucumber (Echinodermata) *Psolus patagonus*, which have been also identified as characteristic species by SIMPER analysis (Supplementary Table St3), contributing the most to the average similarity within the assemblage (25.46%). The Porifera group was also highly represented, although due to the difficulty of identification from photographs, most were not identified at species level. Among the annelids, the most abundant ones were *Perkinsiana* spp. and *Notaulax phaeotaenia*. Pagurids were the most abundant crustaceans found while the most frequent mollusks were *Fisurella* spp. and *Chiton magnificus*. *Arbacia dufresnii* and *Pseudechinus magellanicus* were the most abundant echinoderms.

**Table 1:** List of unique invertebrate taxa or OTUs found within the MAF of *Errina antarctica* located in Angostura Tomms.

Phylum	Species	Phylum	Species
Annelida	*Chaetopterus variopedatus*		*Labidiaster radiosus*
	*Eulalia* sp.		*Loxechinus albus*
	*Notaulax phaeotaenia*		*Odontaster penicillatus*
	*Perkinsiana* sp.		*Ophiacantha* sp.
	Serpulidae		*Pseudechinus magellanicus*
Arthropoda	*Austromegabalanus psittacus*		*Psolus patagonicus*
	*Balanus laevis*	Mollusca	*Argobuccinum pustulosum*
	*Campylonotus vagans*		*Berthella platei*
	*Elminius kingii*		*Calliostoma* sp.
	*Eurypodius latreillii*		*Callochiton puniceus*
	Isopoda		Cerithiopsidae
	*Munida gregaria*		*Chiton magnificus*
	*Nauticaris magellanica*		*Coryphella falklandica*
	*Notobalanus flosculus*		*Diaulula hispida*
	*Pagurus comptus*		Doridoidea
	*Paralomis granulosa*		*Fissurella oriens*
	*Peltarion spinulosum*		*Fisurella* sp.
	Peracarida-Sphaeromatidae		*Fusitriton magellanicus*
Brachiopoda	*Magellania venosa*		*Gargamella immaculata*
	*Terebratella dorsata*		*Itaxia falklandica*
Bryozoa	*Alcyonidium australe*		*Margarella violacea*
	*Beania magellanica*		Muricidae
	*Carbasea ovoidea*		*Nacella flammea*
	*Cellaria* sp.		*Tonicia argyrosticta*
	*Membranipora isabelleana*		*Tonicia disjuncta*
Chordata	*Didemnum studeri*		*Tonicia smithi*
	*Paramolgula gregaria*		*Tritonia challengeriana*
	*Polyzoa opuntia*		*Tritonia odhneri*
	*Sycozoa gaimardi*		*Trophon geversianus*
Cnidaria	*Actinostola chilensis*		*Trophon plicatus*
	*Alcyonium* sp.		*Xymenopsis* sp.
	*Grammaria abietina*		*Zygochlamys patagonica*
	*Halcurias pilatus*	Porifera	*Clathrina fjordica*
	*Primnoella chilensis*		*Haliclona caduca*
Echinodermata	*Anasterias antarctica*		*Haliclona* sp.
	Antedonidae		*Latrunculia ciruela*
	*Arbacia dufresnii*		*Mycale (Aegogropila) magellanica*
	*Asterina fimbriata*		*Oceana spiniphaera*
	*Athyonidium chilensis*		*Raspailia (Raspailia) viminalis*
	*Cosmasterias lurida*		*Scopalina* sp.
	*Florometra magellanica*		*Tedania (Trachytedania)*
	*Gorgonocephalus chilensis*		*Trachytedania spinata*

OTU identified as characteristic of the two biogenic habitats by SIMPER are shown in Supplementary table St3. In both habitats, a small set of species were identified as the main species typifying each assemblage.

## Discussion

This study reports on the presence of the southernmost known living monospecific MAF of *E. antartica* that is also the shallowest stylasterid MAF described worldwide to date. Of the 23 transects carried out in adjacent waters on both sides of the Strait of Magellan, only in Angostura Tomms the species appeared in high density forming a structural complexity typical of MAFs (maximum density = 436 colonies/m^2^). Another reef-like formation of this species densely covering (exceeding 80%) the bottom below 18 m depth was previously recorded further north, in the Copihue Channel -Madre de Dios Archipelago-, by Häussermann and Försterra (2007)^21^. However, in 2013 it was found that this assemblage had lost its structural complexity and only coral rubble was found in the area, for unknown reasons.

Due to the maximum size that *E. antarctica* reaches and the fact that in its development it does not form a true ‘canopy’, assemblages of this species display a more savannah-like appearance than a forest, compared to terrestrial ecosystems. This type of assemblage is considered by the international scientific community as a marine animal forest, since regardless of their size and dominant species, it still substantially increases three-dimensional complexity and has important functional and ecological roles as habitat provider or in nutrient carbon cycling^43^. The term MAF therefore, encompasses different morphologies, from those that do not totally cover the substrate such as sea pens fields, mussel beds, sponge aggregation or stylasterids ‘savannahs’ to dense and continuous covers such as coral reefs.

Few data are available in Patagonian waters related to the presence of MAFs as well as the presence of stylasterid species. This region remains one of the least known and understood marine regions of the world. The work being done in recent years by various NGOs in collaboration with research institutions focused on the observation and study of benthic fauna through underwater explorations is helping to reverse this situation^27,49^. This study is a clear example, as it extends the known geographical distribution of MAFs of *E.antarctica* to the southern tip of the continent in waters around the Strait of Magellan.

Although stylasterids are ubiquitous in marine environments, they have remarkably narrow distributional ranges^44–46^ and they do not give rise to large colonies and savannahs everywhere. This suggests that their formation is conditioned not only by ecological preferences, but also by species ecology^44,47^ and local abiotic factors related to the surrounding water masses^4,7^. The limited dispersal abilities of their larvae^16,48^ together with the dominance of hydrodynamic conditions, high enough to avoid sedimentation on the colonies while providing greater food availability, appear to be key aspects to locally support the development of these monospecific savannahs. These specific ecological and environmental conditions that affect the presence of these species at high densities would explain the low number of MAFs of these colonial calcifying hydrozoans described worldwide^11,16,17^.

The MAF of *E. antarctica* in Angostura Tomms with coverages reaching up to 28.5% of the substrate from 1.23 m to, at least, 33 m depth is, to our knowledge, the shallowest stylasterid savannah described to date. Although there are records of this eurybathic species in deep areas of the Falkland Islands and Burdwood Bank in the Scotia Arc up to 771 m^20^ and previous records of similar aggregations have also occurred at shallow depths in Patagonian waters, they were always below 18 m^21^. Other aggregations of other species of stylaterids exist in shallow water, as is the case of *Stylaster californicus* aggregations in California or the patchy populations of *E. novaezelandiae* at depths > 20–3 m in the New Zealand Fjord^16^, but not as shallow as the one described in this study. In fact, these are isolated records, since this group of hydrozoans is most abundant between 200–1200 m—although they can be found from the intertidal zone to depths up to 2800 m^44^—and only a few species of Stylasteridae are known to inhabit shallow waters^45^.

The reasons for the origin or formation of these localized shallow MAFs in certain shallow areas are unclear. Lindner et al.^18^ inferred that all stylasterids derive from a common ancestor that originated and diversified in the deep sea, after which invasions of shallow environments where they currently dwell, occurred. However, the presence of eurybathic stylasterids in both the deep-sea and shallow-water fjords, seems to be rather a recent extension of their distribution range as a consequence of a phenomenon known as deep water emergence^18,49^. This phenomenon, which has been described for different species in fjords in different regions of the world, such as the antipatharians of New Zealand^50^, the stony coral *Lophelia pertusa* from Norway^51^ and *Errina novaezelandiae* from New Zealand^16^, describes the fact that species that are usually restricted to deep water throughout much of their distribution are also common and locally abundant in specific shallow areas, in this case, in a fjord system. A wide variety of causes have been proposed to explain this phenomenon and, although none of them are able to explain it in the different parts where it occurs, the factors most frequently considered are low light levels, similar low temperatures at the surface and in deep water, and low sedimentation rates^52^. Specifically in Chile, Hausserman et al.^52^ found this pattern in the fjords of Chilean Patagonia for 32 species and six genera. Of these, although the largest number were Echinodermata, 10 species and four genera were Cnidaria and included *E. antarctica*, previously mentioned as a deep-water emergent species by Häussermann and Försterra^21^.

Another characteristic of the MAF of *E. antarctica* in Angostura Tomms is that various types of growth were recorded, from two-dimensional fan-shaped to densely branched multidimensional colonies,—as previously described by Häussermann and Försterra^21^ for this same species, and by Salvati et al.^11^ for another species of the same genus—, with maximum length sizes around 16 cm. Larger colonies of *E. antarctica* have been described by these same authors for the Copihue Channel, where colonies reached 40 cm in diameter. The highly right-skewed unimodal size frequency distribution with a larger proportion of small colonies (99% < 10 cm and only 1% of the colonies being 10–20 cm) in Angostura Tomms suggests a predominance of recruitment processes and a limited growth or higher mortality of older colonies. This population structure with small colonies as the most abundant and larger colonies as the least abundant have been found in other aggregations of species of this genus, such as the populations structure of *E. novaezelandiae* in New Zealand (50% < 10 cm; 30%: 10–20 cm; and 15–20% > 20 cm)^16^ or *E. aspera* in Messina Strait^11^. Information on the biology and ecology of *E. antarctica* is not yet available but estimates of the annual growth rate of *E. novaezelandiae*, another emerging species of red coral from a fjord system and which has a similar type of colony growth, at least the flat dimensional shape, have been made. For this species, the average annual growth is 1–2 cm/year in height and/or width, which can reach 6.8 cm/year in some isolated colonies, and the net growth is 0.7 cm/year, because of partial mortality which occurs mainly in larger colonies due to diving activities and the dislodgement of whole colonies from the substrate, rather than their fragmentation or breakage^16^. Assuming similar slow growth and based on these values, we estimate that if the average growth is about 1.5 cm/year, only 0.24% of the colonies of *E. antarctica* in Angostura Tooms would reach 10 years of age and if we consider the net value of 0.7 cm/year, the percentage of colonies older than 10 years would rise to 4.4%, with a maximum age of 22 years old. If we were to consider the maximum growth without partial mortality (6.8 cm/year), no colony would be more than 2 years old. Another hypothesis that explains the absence of large colonies in larger proportions is that the hydrodynamic conditions of the area strongly constrain their growth because the colonies are not able to withstand the strong currents and their apical branches break, as has been reported by Salvati et al.^11^ for *E. aspera* in the Messina Strait. In this study, in view of the results obtained, we suggest two different hypotheses, which are that the Angostura Tomms MAFs could correspond to (a) a recent colonization of a space, extending its distribution range towards shallower areas, as a consequence of some hydrographic event. The low larval dispersal would lead to the formation of a dense savannah at a stage of the ecological succession directed towards increasing colony sizes, greater occupation of space and ecological climax; (b) a savannah formed at the limit of the species’ distribution in which the environmental conditions are not optimal for the major development of the colonies, maintaining the characteristics of a stable savannah without evolving or generating changes in its population structure.

The occurrence of habitats structured by three-dimensional sessile invertebrates considered ecosystem engineers^53^ such as E*. antarctica* but also *C. variopedatus*, would explain the higher values of species richness and diversity found in the area, as demonstrated through this study. Our results are consistent with previous studies that note that the presence of habitat-forming species increases habitat complexity and therefore, increases species richness and alpha biodiversity^3,6,54,55^. These habitat-formers serve as settlement substrate for epifauna and refuge from predation, and also facilitates encounters with other prey that serve as feeding opportunities, lastly enhancing biodiversity and sustaining high levels of ecosystem functioning^6,10,56,57^. Focusing on the *E. antarctica* savannah, a list of 88 OTUs associated with the MAF of *E. antarctica* has been compiled from the photos although we assume that this is, possibly, an underestimate due to the difficulty of visualizing species hidden among branches and identifying species from photos once found. However, and despite this difficulty, we can confirm that many of the most conspicuous representatives recorded in the *E. antarctica* savannah in Angostura Tomms have been found associated with aggregations of this species in other areas of Patagonia^2,30^, as is the case of other Cnidaria such as *Actinostola chilensis*, Bryozoa such as *Carbasea ovoidea*, Echinodermata such as *Arbacia dufresneij*, *Gorgonocephalus chilensis* and *Labidiaster radiosus,* Crustacea such as *Pagurus comptus* and *Paralomis granulosa*, Mollusca such as *Calliostoma* sp., Polychaete such as *C. variopedatus* and Porifera such as *Haliclona* sp. and other not identified sponges.

Most of this fauna characteristic of the *E. antarctica* savannah, as well as the most abundant species in the rest of habitats found in the study area, were sessile, while crawler species were less abundant. This could be related to the harsh environmental conditions of the Strait of Magellan that imply an intermediate–high level of environmental stress and that may affect the presence of mobile consumers, which tend not to be abundant in relatively stressful habitats^58,59^.

This unique and diverse benthic assemblage makes Angostura Tomms an area of great ecological importance. Maintaining biodiversity is important for maintaining ecosystem functioning^26,60^, since highly diverse MAFs would be more buffered and resilient environments to disturbances than those with fewer functional redundancies^61^. A higher number of species results in a higher number of functionally similar species and then, in a higher number of possible responses to perturbations. For this reason, an in-depth study of the functionality of this specific assemblage would serve to reinforce and verify this theory. Therefore, a useful next step in the research would be to use the biological traits analysis (BTA) approach^62^ to acquire a deeper view of its functional diversity, research that would benefit from studying a larger number of samples.

Among the great diversity of ecosystem functions, stylasterids, like other perennial MAFs, has fundamental functions for the integration of the compartments of the marine ecosystem based on the exchange between benthos and plankton^63^. Due to their feeding, as suspension feeders that capture large quantities of particles from the water column, and their reproduction, mostly as brooders of planulae that they release into the water, they have a fundamental role in the transfer of matter and energy from plankton to benthos and vice versa, enhancing the benthic-pelagic coupling^2,47,64^. However, despite these ecosystem’ functions and forming more buffer habitats against environmental changes, the life-history characteristics of the colonies of this group of hydrozoans make them more vulnerable to changes derived from anthropogenic activities than seasonal hydroids that have the ability to enter dormancy^65^, hence they are considered sentinel species. The presumed slow growth and extended longevity of *E. antarctica* as well as its structure population in Angostura Tooms characterized by skewed distributions dominated by small colonies with only around 1% of the colonies classified as medium-sized and no large-sized colonies, probably contributing disproportionately to reproduction (e.g. 16, 66), make it likely that maintenance of this MAF as well as recovery of the species if it suffers any damage, will take decades if it occurs at all.

Angostura Tomms is located in the Kawésqar National Reserve (KNR). This is the 6th largest marine protected area (MPA) in the country, with 2,628,429 ha (Decree 6 of 26 January 2018, Chile). According to the Convention for the Protection of the Flora, Fauna and Natural Scenic Beauty of America (Washington, 1940), to which Chile is a signatory, a National Reserves denotes the use of the natural wealth to be compatible with the conservation of natural resources, guaranteeing that the flora and fauna receive the protection that maintains the purposes for which these reserves were created. Despite it is currently a lightly impacted marine area and harbors unique ecosystems such as the MAF of *E. antarctica* that is the subject of this study, it allows the development of multiple uses (e.g. navigation, fishing, aquaculture) in its waters. The salmon farming is one of the most detrimental to benthic communities^67^. Currently, 57 salmon aquaculture concessions are granted within the National Reserve and 132 new concessions are currently being requested^68^, which clearly threatens the benthic communities and the biodiversity of its waters. In this regard, Martínez and Paredes (2020)^68^ point out that the “development of salmon farming inside National Reserves” is at least questionable both in environmental and legal terms, to the extent that it does not constitute, strictly speaking “use of the natural wealth of such protected areas”, since the salmonid species are exotic.

The need for conservation actions to ensure the survival of the richness of species and biodiversity associated with benthic communities and specifically, the existing MAFs in Chilean Patagonian waters, must include concrete and effective tools, such as the designation of no-take zones, the development of management plans that clearly defines the role of the different actors, the recategorization of the National Reserves into Marine or National Parks, and the removal of aquaculture concessions from its protected waters in order to ensure the conservation of biodiversity in the long term.

Our study highlights the importance of the benthic communities existing in this MPA, highlighting the need to act actively to ensure their maintenance.

### Supplementary Information

Supplementary Video 1.

Supplementary Video 2.

Supplementary Information.

## Data Availability

The datasets used and/or analysed during the current study are available from the corresponding author on reasonable request.
