# Supplementary material for: The southernmost Errina antarctica hydrocoral savannah in Patagonian waters
Source: Sci Rep. 2024 Apr 26;14:9579. doi: 10.1038/s41598-024-60207-2 (PMC11053091; doi:10.1038/s41598-024-60207-2)
Supplement: Supplementary file 3 — Supplementary Information. [file 41598_2024_60207_MOESM3_ESM.docx]

SUPPLEMENTARY MATERIAL

SUPPLEMENTARY FIGURE

**Figure Sf1.** Species accumulation curves (SAC) with increasing number of replicates of the 7 habitats types sampled. The boxplots reflect the rate of new OTUs/species under continuous sampling, generating by random selection of sites, showing the mean and confidence intervals. In color, the species richness (S) of each habitat is indicated for the same number of samples (in light blue, N=3 and dark blue, N= 8).

**
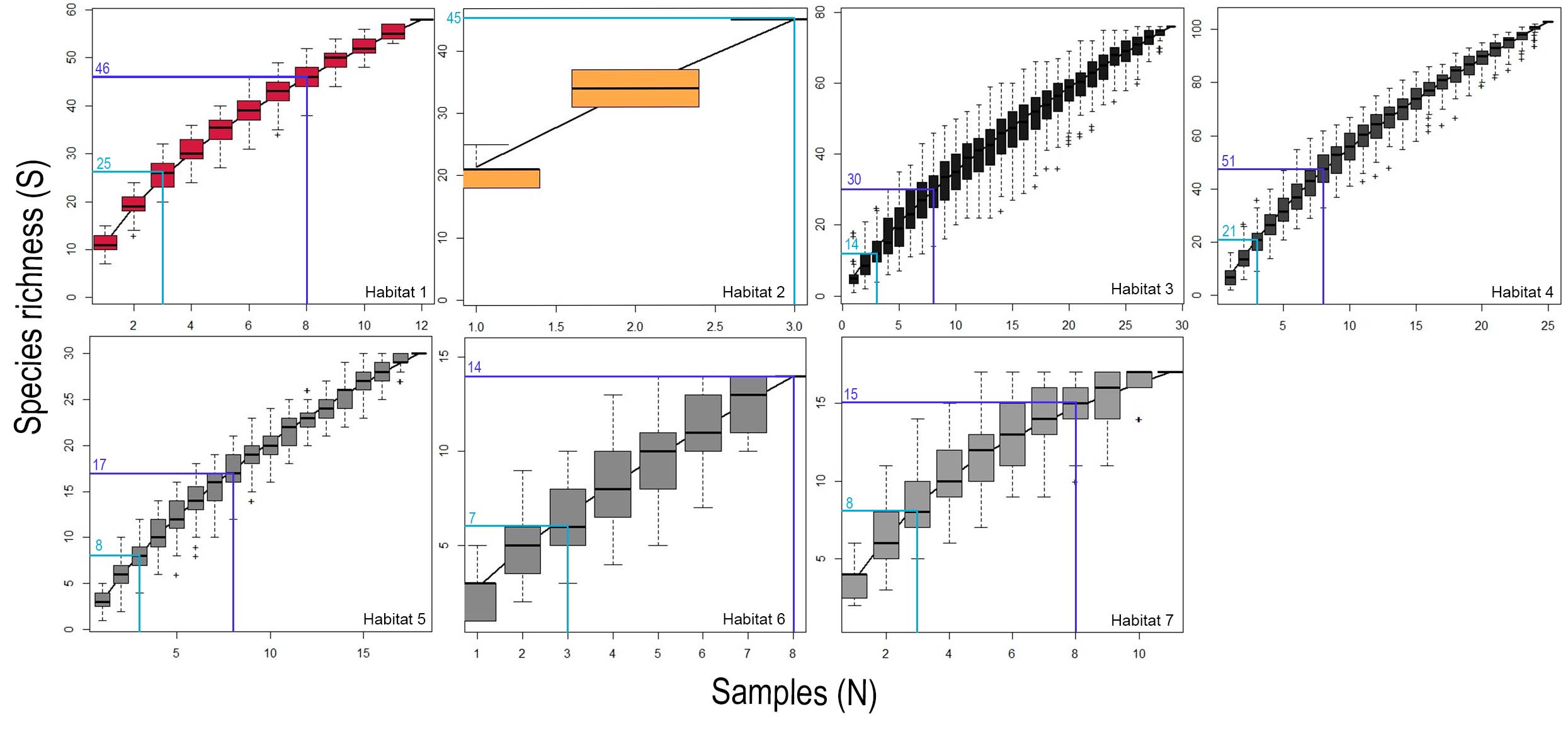
**

SUPPLEMENTARY TABLES

**Table St1.** Number of photographs per transect and depth taken in the different expeditions. The number of photos in each transect and at each depth in which at least one colony of *Errina antarctica* was found is shown in parentheses.

| TRANSECT | EXPEDITION | Z= 5 m | Z= 10 m | Z= 15 m | Z= 20 m | Z= 25 m |
| --- | --- | --- | --- | --- | --- | --- |
| TR01 | April 2021 | 3 | 4 | 4 | 4 | 0 |
| TR02 | April 2021 | 5 | 4 | 4 | 4 | 4 (1) |
| TR03 | April 2021 | 5 | 4 | 4 | 4 | 4 |
| TR04 | April 2021 | 4 | 5 | 0 | 0 | 0 |
| TR05 | April 2021 | 5 | 4 | 4 (4) | 4 (4) | 4 (1) |
| TR06 | April 2021 | 4 | 4 | 4 | 4 | 4 |
| TR07 | April 2021 | 5 | 5 | 5 | 0 | 0 |
| TR08 | April 2021 | 0 | 0 | 4 | 0 | 0 |
| TR09 | July 2021 | 4 | 4 | 4 | 0 | 0 |
| TR10 | July 2021 | 4 | 4 | 4 | 0 | 0 |
| TR11 | July 2021 | 4 | 4 | 4 | 0 | 0 |
| TR12 | July 2021 | 4 | 4 | 4 | 0 | 0 |
| TR13 | July 2021 | 4 | 4 | 4 (1) | 0 | 0 |
| TR14 | July 2021 | 4 | 4 | 4 | 0 | 0 |
| TR15 | July 2021 | 4 | 4 | 4 | 0 | 0 |
| TR16 | July 2021 | 4 | 4 | 4 | 0 | 0 |
| TR17 | July 2021 | 4 | 4 | 4 | 0 | 0 |
| TR18 | July 2021 | 4 | 4 | 4 | 0 | 0 |
| TR19 | July 2021 | 4 | 4 | 4 | 0 | 0 |
| TR20 | July 2021 | 4 | 4 | 0 | 0 | 0 |
| TR21 | July 2021 | 4 | 4 | 4 | 0 | 0 |
| TR22 | July 2021 | 4 | 4 | 4 | 0 | 0 |
| TR23 | August 2022 | 3 (3) | 1 (1) | 2 (2) | 1 (1) | 0 |

**Table St2.** Average between-group dissimilarities (%) between the habitats for the taxonomic composition of invertebrates, as result of the SIMPER analysis.

| HABITAT | AVERAGE  BETWEEN-GROUP DIDSIMILARITY (%) |
| --- | --- |
| Hab1- Hab2 | 92.41 |
| Hab1- Hab3 | 96.87 |
| Hab1- Hab4 | 97.06 |
| Hab1- Hab5 | 95.79 |
| Hab1- Hab6 | 99.67 |
| Hab1- Hab7 | 95.87 |
| Hab2- Hab3 | 95.30 |
| Hab2- Hab4 | 92.05 |
| Hab2- Hab5 | 89.23 |
| Hab2- Hab6 | 84.06 |
| Hab2- Hab7 | 90.98 |
| Hab3- Hab4 | 96.23 |
| Hab3- Hab5 | 93.61 |
| Hab3- Hab6 | 96.69 |
| Hab3- Hab7 | 96.61 |
| Hab4- Hab5 | 94.86 |
| Hab4- Hab6 | 98.74 |
| Hab4- Hab7 | 98.34 |
| Hab5- Hab6 | 98.79 |
| Hab5- Hab7 | 95.95 |
| Hab6- Hab7 | 95.28 |

**Table St3**. Characteristics operational taxonomic units (OTU) and their contribution to the two biogenic habitats studied (Hab1-Rocky bottom with *Errina antarctica*, and Hab2-Rocky bottom with *Chaetopterus variopedatus*) as identified by SIMPER analyses.

| HABITAT | AVERAGE  SIMILARITY | SPECIES  (Contribution 95%) | SPECIES CONTRIBUTION  (Cumulative %) |
| --- | --- | --- | --- |
| 1 | 25.46 | *Errina antarctica*  *Psolus patagonicus*  *Balanus laevis Elminius kingii* | 65.44  75.13  84.51  92.55 |
|  |  |  |  |
| 2 | 32.95 | *Chaetopterus variopedatus*  *Balanus laevis* Hidrozoa  *Magellania venosa*  Porifera (indeterminate)  *Primnoella chilensis* | 45.66  72.66  77.79  82.35  89.11  91.09 |
|  |  |  |  |

SUPPLEMENTARY VIDEOS

**Video Sv1:** Scuba-diving video footage showing the MAF of *Errina antarctica* from 28 to 23 m depth.

**Video Sv2:** Video-capture of a ROV transect showing the MAF of *Errina antarctica* at around 44-45 m depth.
